# Analysis of polaron pair lifetime dynamics and secondary processes in exciplex driven TADF OLEDs using organic magnetic field effects

**DOI:** 10.1038/s41598-024-82060-z

**Published:** 2024-12-17

**Authors:** Annika Morgenstern, Dominik Weber, Lukas Hertling, Konstantin Gabel, Ulrich T. Schwarz, Daniel Schondelmaier, Dietrich R. T. Zahn, Georgeta Salvan

**Affiliations:** 1 Semiconductor Physics, Institute of Physics, Chemnitz University of Technology, 09126 Chemnitz, Germany; 2 Nanotechnology, University of Applied Sciences Zwickau, Physical Engineering and Computer Science, 08056 Zwickau, Germany; 3 Experimental Sensor Science, Institute of Physics, Chemnitz University of Technology, 09126 Chemnitz, Germany; 4 Center of Materials, Architectures and Integration of Nanomembranes (MAIN), Chemnitz University of Technology, 09126 Chemnitz, Germany

**Keywords:** Thermally activated delayed fluorescence (TADF), Exciplex, Organic magnetic field effects, Polaron-pair dynamics, Triplet-triplet annihilation, Triplet-polaron interaction, K-fold cross-validation, Organic LEDs, Magneto-optics, Electronic devices

## Abstract

Magnetic field effects (MFEs) in thermally activated delayed fluorescence (TADF) materials have been shown to influence the reverse intersystem crossing (RISC) and to impact on electroluminescence (EL) and conductivity. Here, we present a novel model combining Cole–Cole and Lorentzian functions to describe low and high magnetic field effects originating from hyperfine coupling, the $\Delta$g mechanism, and triplet processes. We applied this approach to organic light-emitting devices of third generation based on tris(4-carbazoyl-9-ylphenyl)amine (TCTA) and 2,2′,2″-(1,3,5-benzinetriyl)-tris(1-phenyl-1-H-benzimidazole) (TPBi), exhibiting blue emission, to unravel their loss mechanisms. The quality of the regression function was evaluated using k-fold cross-validation. The scoring was compared to various alternative fitting functions, which were previously proposed in literature. Density functional theory calculations, photoluminescence, and electroluminescence studies validated the formation of a TADF exciplex system. Furthermore, we propose successful encapsulation using a semi-permeable polymer, showing promising results for magnetic field sensing applications on arbitrary geometry. This study provides insights into the origin of magnetic field effects in exciplex-TADF materials, with potential applications in optoelectronic devices and sensing technologies.

## Introduction

Organic light emitting diodes are nowadays widely used and have become indispensable in everyday technology, such as smartphone displays, high-quality TVs, monitors, and smartwatches^1^. However, ongoing research into OLED materials and architectures continues to push the boundaries, paving the way for new applications like flat-panel displays^1^, sensor applications^1^ and point-of-care devices in medical applications^2^. Several approaches have been tested to achieve high internal quantum efficiency bypassing the spin-statistics of generated excitons^3–5^. One approach is based on the integration of heavy metal complexes, where the emission is governed by phosphorescence (triplet ($\mathrm {T_1}$) state $\rightarrow$ singlet ground state ($\mathrm {S_0}$)), allowing to overcome the quantum statistical limitation of $25\%$ internal quantum efficiency^6,7^. Another approach, introduced by Adachi et al.^8^, is based on materials that exhibit a small intrinsic $\Delta \textrm{E}_{\textrm{ST}}$ gap between $\mathrm {T_1}$ (or $\mathrm {T_2}$^9^) and the excited singlet ($\mathrm {S_1}$) state^9^. When this energy difference is in the range of a few meV, up-conversion takes place from $\mathrm {T_1}$ to $\mathrm {S_1}$, leading to thermally activated delayed fluorescence (TADF)^10,11^. For exciplex-based TADF emitters, it is well established that this gap is minimal and can even approach zero^11,12^. The radiative emission takes place along with the $\mathrm {S_1}$ $\rightarrow$ $\mathrm {S_0}$ transition^5^ and TADF thus enhances the internal quantum efficiency. Besides the excitonic-TADF materials^13^, which exhibit an intrinsic $\Delta \textrm{E}_{\textrm{ST}}$ (cf. ref.^14^), excited complex (exciplex)-TADF materials currently attract significant attention, because their $\Delta \textrm{E}_{\textrm{ST}}$ can be tuned upon the choice of the donor and acceptor molecules forming the charge-transfer complex. Here the new states ($\mathrm {EX_1}$ and $\mathrm {EX_3}$) arise from the overlap of the electron and hole wavefunction of two separated acceptor and donor molecules^11^. Especially in the field of organic spintronics, this material class seems to be highly valuable, as they exhibit promising reverse-intersystem crossing (RISC) rates and show high sensitivity to low magnetic fields^10,11,15–17^. The sensitivity to magnetic fields in organic materials is generally termed as magnetic field effect (MFE) and can be observed experimentally in organic magnetoresistance (OMAR), organic magnetoconductance (OMC), or organic magnetoelectroluminescence (MEL). Most MFE arise from the influence of an external magnetic field on the spin mixing between singlet and triplet states in polaron pairs (PPs), bipolarons, and exciplexes (or excitons) generated by electrically injected charges. In the absence of an external magnetic field, this spin mixing is primarily governed by the hyperfine interaction, the $\Delta$g mechanism^18,19^, and/or triplet-polaron interactions^20^. However, since these interactions are highly sensitive to external magnetic fields, their behavior is altered when such a field is present. Consequently, organic magnetic field effects can be used not only to manipulate the device response, but also to characterize the spin dynamics of OLEDs and, if necessary, to understand the degradation mechanisms of OLED devices non-destructively^21,22^. By fitting the lineshape of the magnetoelectroluminescence, which is determined as follows $\mathrm {MEL = [EL(B) - EL(B = 0)]/ EL(B = 0)}$, with a Cole–Cole fitting function^23^ it is possible to take the dispersive lifetime of the injected charge carriers in organic semiconductors into account. By this approach, the mean polaron-pair lifetime and the dispersive parameter $\alpha$ can be extracted. The dispersive parameter $\alpha$ is related to the degree of disorder and its value decreases with the device degradation^22^. Furthermore, different loss mechanisms would influence the MEL broadening in different manner. The low-field effects (LFE) are those effects that dominate the response of the device and the lineshape of the MFE curves at magnetic fields below $|20 |$ mT, i.e. hyperfine interaction and $\Delta$g mechanism. It has been suggested that the full-width at half maximum (FWHM) of the MEL curves will be larger than 4 mT, if the $\Delta$g mechanism dominates over the hyperfine interaction^12,22^. Since the $\Delta$g mechanism originates from slight differences in the Larmor frequencies of electrons and holes, an out-of-phase behavior is expected for the precession of the spin of the positive and negative polarons around the hyperfine field. Eventually, after some time the precession reaches a steady-state condition. For devices in the steady state, a restored Lorentzian line shape of the MFE(*B*) is usually experimentally observed^24^. Additional contributions at higher magnetic fields can be assigned to triplet processes, e.g. triplet-polaron interactions (TPI)^20^ or triplet-triplet annihilation (TTA)^25^. When studying the MFEs in exciplex-TADF systems, the donor and acceptor molecules were often chosen such that the energetic distance between the highest occupied molecular orbital of the donor $\text {HOMO}_\text {donor}$ and the lowest unoccupied molecular orbital of the acceptor $\text {LUMO}_\text {acceptor}$ was small, since it was found to have crucial impact on the MFE strength^26^. Nevertheless, a large $\text {HOMO}_\text {donor}-\text {LUMO}_\text {acceptor}$ gap is required when designing blue-light emitting OLEDs, e.g. for full-color display applications^27^. We recently demonstrated that the combination of tris(4-carbazoyl-9-ylphenyl)amine (TCTA) as donor and 2,2’,2”-(1,3,5-benzinetriyl)-tris(1-phenyl-1-H-benzimidazole) (TPBi) as acceptor yields to the formation of exciplexes exhibiting blue-light emission in the fabricated third-generation OLEDs as well as sensitivity to external magnetic fields^28^. In this study, we employ a novel approach to fit the lineshape of the MEL response, utilizing a combination of Cole–Cole and Lorentzian terms to separately extract parameters of interest for LFE and HFE. The quality and transferability of the fitting function are evaluated using the fivefold cross-validation algorithm, which has been largely used in machine learning applications. We compared the scoring factor of our fit to several other fitting functions used in literature^18,20,22^. This method allows us to propose a universal fitting function suitable for materials with a strong dependence on triplet processes and dispersive character of the injected charge carriers. We compared the MFEs of numerous devices fabricated using various OLED architectures and deposition techniques, including spin coating and vapor deposition. Additionally, we explored the impact of two different encapsulants, one of which shows high potential for future gas sensing applications^29^ due to its semi-permeability^30^ or in the realization of magnetic field sensor arrays on arbitrary geometry.

## Results and discussion

**Fig. 1:**
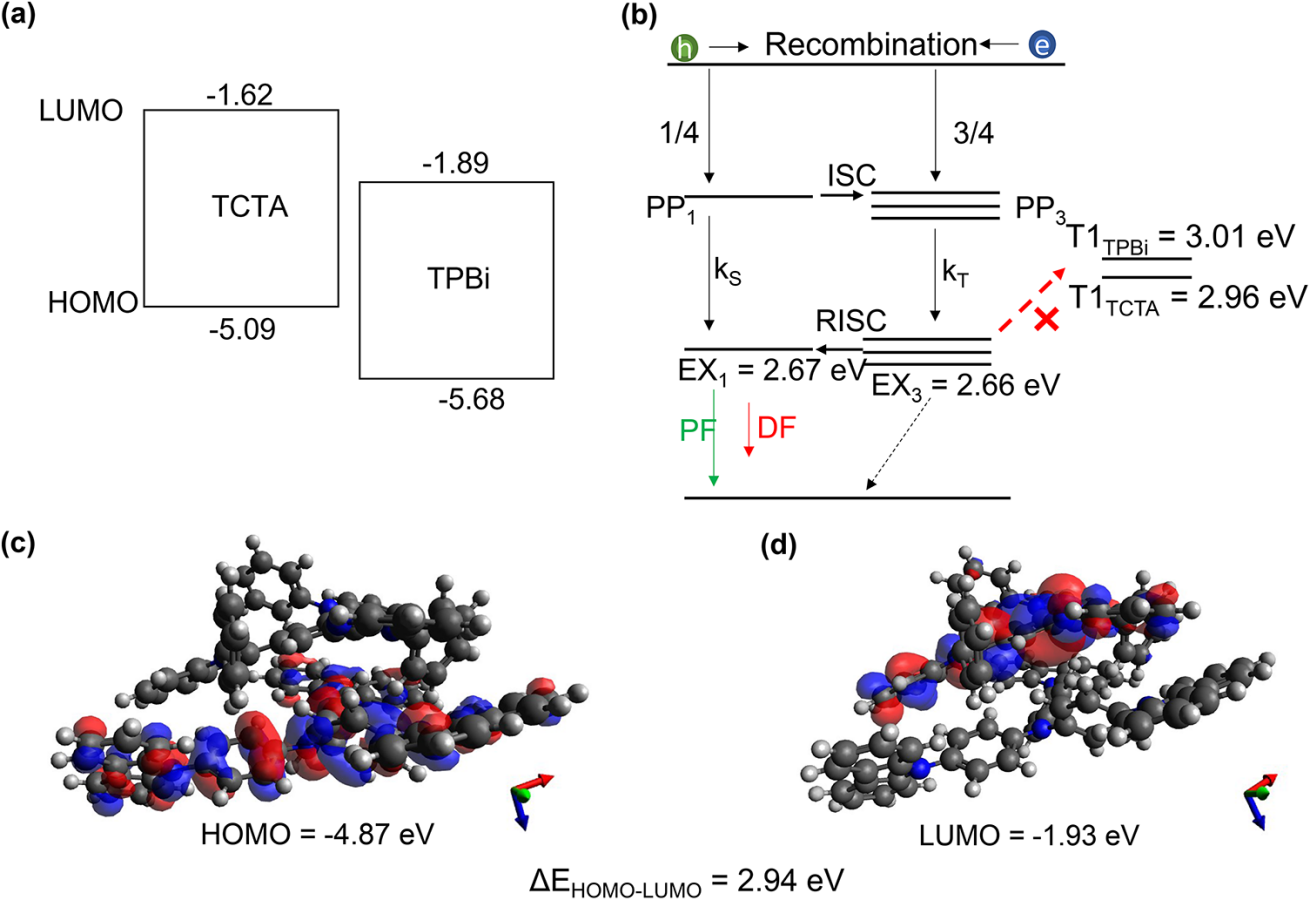
(**a**) HOMO- LUMO positions relative to the vacuum level and energy alignment in the TCTA (D) and TPBi (A) system determined by DFT calculations, (**b**) Schematic Jablonski diagram of the TCTA-TPBi complex, illustrating the energy levels as determined by DFT calculations. Upon applying a constant voltage, electrons (e) and holes (h) are injected into the system. Spin statistics dictate the formation of $25\%$ singlet ($\text {PP}_1$) and $75\%$ triplet ($\text {PP}_3$) polaron pairs. Both prompt fluorescence (PF) and delayed fluorescence (DF) occur from the $\mathrm {EX_1}$ $\rightarrow$ $\mathrm {S_0}$ transition. RISC is expected to occur between the $\mathrm {EX_3}$ $\rightarrow$ $\mathrm {EX_1}$ state, (**c**) and (**d**) depict the molecular structure of the TCTA-TPBi dimer, highlighting the HOMO and LUMO orbitals, respectively.

**Fig. 2:**
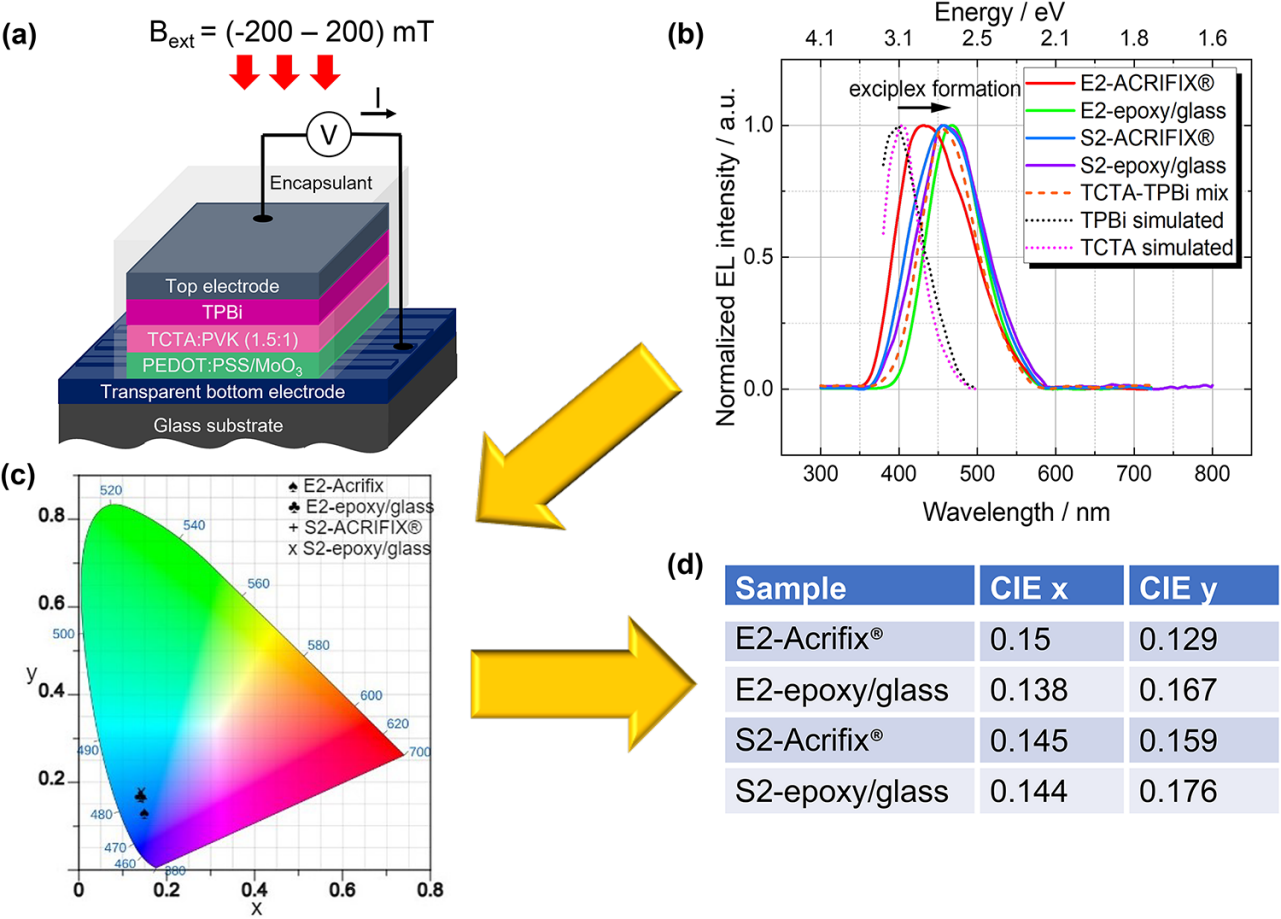
(**a**) Schematic layer stack of the fabricated devices, (**b**) Electroluminescence simulated by SETFOS software^42^ from the measured photoluminescence spectra for the single compounds compared to the measured response for the mixed layer exhibiting a clear redshift. Additionally, a clear red shift can be observed for the different layer stacks used in this work, which is a clear indicator for exciplex formation. (**c**) CIE chromaticity diagram for all devices produced in this work, with a significant outliner for the electroluminescence position for the E2-ACRIFIX$\circledR$ device. (**d**) CIE x and CIE y for values for the spectra shown in (**b**) and the CIE chromaticity diagram in (**c**).

To ensure that the TADF material combination exhibits exciplex formation, a combination of donor and acceptor molecules, namely TCTA as donor and TPBi as acceptor, was chosen (see Fig. 1a for the HOMO and LUMO levels). Additionally, polyvinylcarbazole (PVK) was used in a blend with TCTA (1:1.5) to enhance the device stability. As previously reported, PVK is known to influence the hole mobility and consequently the charge carrier balance. Consequently, the external quantum efficiency can be enhanced^28,31,32^. The used layer stack is depicted in Fig. 2a. Two different types of devices were fabricated, where the TCTA:PVK blend was always deposited from solution, while TPBi was either evaporated by physical vapor deposition or spin-coated from solution. For more details, we refer to our previous work^28^ (cf. Fig. 2.3 Devices E2 and S2 as well as section 2) and the Experimental details section of this work. For consistency, we use the same terms as in ref.^28^, E2 and S2, to describe the different device layer stacks. Furthermore, both types of devices were encapsulated by ACRIFIX$\circledR$ or epoxy resin and a glass slide, respectively. For the sake of simplicity, we will use the terms *epoxy/glass* instead of *epoxy resin and glass slide* throughout the manuscript. The HOMO and LUMO levels for TCTA and TPBi, as obtained from the DFT calculations, are shown in Fig. 1a. The energy values are relative to the vacuum level. In our previous work^28^, the HOMO and LUMO levels provided by the supplier Ossila^33,34^ were considered. Those values were likely obtained using a combination of optical absorption and photoemission spectroscopy, or cyclic voltammetry (CV). It is widely accepted that the optical gap determined by optical spectroscopic methods is lower than the transport gap in organic materials, due to the exciton binding energy, see e.g. ref.^35^. The exciton binding energy, which is typically in the range of a few hundred meV–1 eV in organic semiconductors, can also be responsible for a significant difference when comparing these results to DFT calculations^36,37^. Additionally, measurements in solution (such as CV) are expected to produce even smaller HOMO-LUMO gaps compared to isolated molecules or molecular solids depending on the molecular packing geometry^35,38^. We will, therefore, in the following, focus on the discussion of the energy of the HOMO and LUMO levels obtained from DFT calculations. Even though this method cannot precisely predict the absolute values of HOMO and LUMO energy^39^, it predicts the singlet and triplet energies with sufficient accuracy.

### Density functional theory calculations

The lowest singlet and triplet level for the single compounds as well as for the D-A-exciplex were calculated using DFT calculations based on O3LYP hybrid functional using Orca 5.0 software^40^. We found three dimer configurations exhibiting very similar values, of which only one example is shown here. From the values in Table 1 the singlet excitation energies agree reasonably with those from the experiment (cf. Fig. 2b). Furthermore, the $\Delta \textrm{E}_{\textrm{ST}}$ for the pristine molecules is in the range of $(0.2 - 0.5)$ eV, which is in agreement with previously reported values for TCTA and TPBi^41^. According to the overlap of the electron and hole wavefunctions, new energetic states, namely $\mathrm {EX_1}$ and $\mathrm {EX_3}$, can be formed with a very small $\Delta \mathrm {E_{EX_1-EX_3}}$ raising the possibility of reverse intersystem crossing at room temperature. The $\mathrm {EX_3}$ level (cf. Fig. 1b) is lower than the $\mathrm {T_{1-TCTA}}$ and $\mathrm {T_{1-TPBi}}$ level, which is why the D and A triplet levels cannot serve as non-radiative recombination channels and the exciplex emission is very efficient^11^. As shown in Fig. 1c and d, the HOMO and LUMO levels are determined by the individual molecules in the dimer, with the HOMO localized on the TCTA molecule and the LUMO on the TPBi molecule. It should be noted that the specific mixture within the solution can lead to less efficient dimer configurations, where the $\Delta \mathrm {E_{EX_1-EX_3}}$ may increase, reducing the likelihood of RISC to happen.

**Table 1:** Calculated values for the lowest energy level of the dimer configuration (exciplex) using DFT calculations.

	TPBi	TCTA	Dimer (Exciplex)
LUMO/eV	− 1.89	− 1.62	− 1.93
HOMO/eV	− 5.68	− 5.09	− 4.87
$\Delta \text {E}_\text {HOMO-LUMO}$/eV	3.79	3.48	2.94
Singlet excitation (DFT/ Experiment)/eV	3.48/ **3.14**	3.20/ **3.09**	2.67/ **2.69**
Triplet excitation/eV	3.01	2.96	2.66
$\Delta \mathrm {E_{ST}}$ /eV	0.47	0.24	-
$\Delta \mathrm {E_{EX_1-EX_3}}$/eV	-	-	0.01

The singlet excitation energy for the pristine acceptor and donor molecules and for the dimer configuration is compared to the results obtained by the electroluminescence measurements (cf. Fig. 2b). The respective $\Delta \mathrm {E_{ST}}$ was obtained for the pristine molecules and dimer configuration ($\Delta \mathrm {E_{EX_1-EX_3}}$). Further, the HOMO and LUMO levels as well as the HOMO-LUMO gap were calculated for the pristine molecules and the dimer configuration by DFT calculations.

### Optoelectronic characterization

**Fig. 3:**
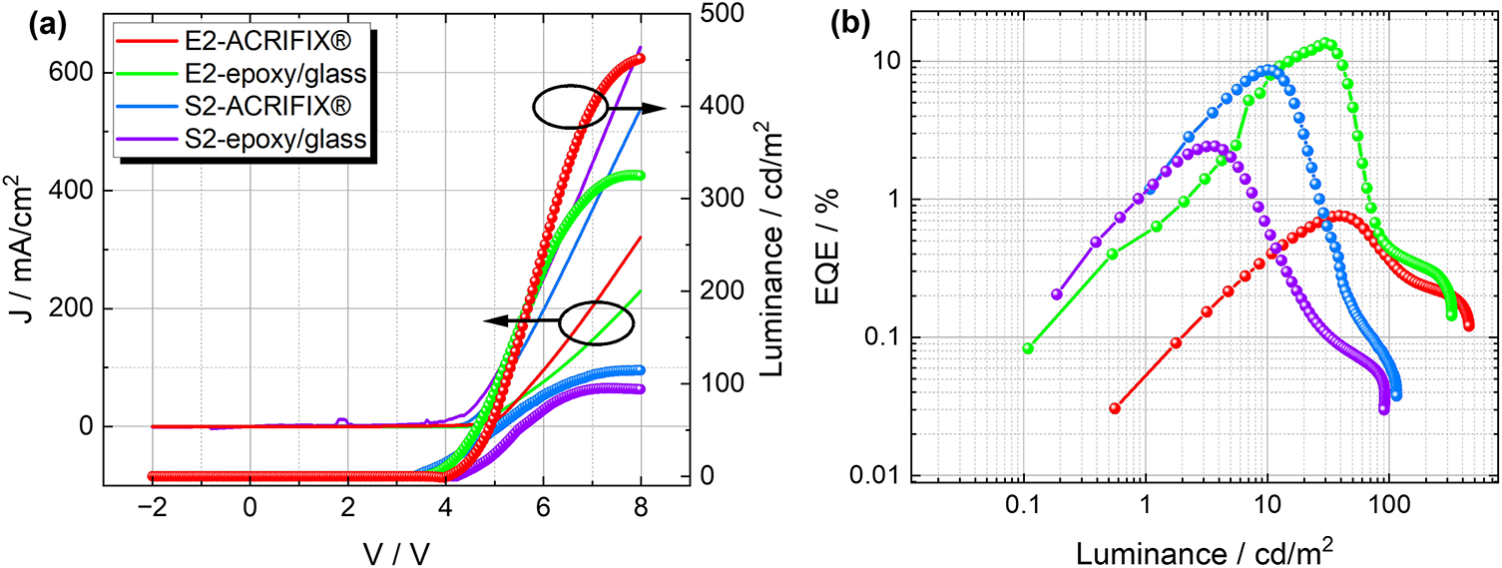
(**a**) Current density (symbols) and luminance (continuous lines) as a function of the applied bias voltage. The linearity observed in the current density indicates a stable charge injection, which is crucial for device performance, and (**b**) the EQE calculated for the devices E2 and S2 encapsulated by ACRIFIX$\circledR$ or epoxy/glass, showing a strong efficiency roll-off.

In Fig. 3a the room temperature current density and the respective luminance as a function of applied bias voltage are depicted. All devices show common diode characteristics, where no strong leakage currents (substantial current density at negative applied voltage) were observed. By taking a look at Fig. 3b similar values were obtained for the external quantum efficiency (EQE) as in our previous work (cf. ref.^28^). The max. EQE of $(13.52 \pm 3.07)\%$ was observed for the E2-glass/epoxy sample, which is very reasonable for CIE (*Commission internationale de l’éclairage*) chromaticity values of $(0.138;\,0.167)$^43^. The exact values for the $\mathrm {{EQE}_{max}}$ for all devices can be found in Table S1. Unfortunately, the devices show a strong efficiency roll-off, which can be attributed to the existence of certain loss mechanisms, e.g. triple-triplet-annihilation or triplet-polaron-interaction^44^. Furthermore, a slight discrepancy was observed for the different encapsulation materials in both cases, which might be caused by unintentional doping of the OLED active layer during the deposition and/or UV illumination of the encapsulant. The absorption spectra (cf. Fig. S1) show a clear difference in the onset as well as in the line shape. As can be seen, only the UV-illuminated ACRIFIX$\circledR$ samples show a clear onset of absorption. Rashidian^45^ reported a significant change in the dielectric properties when treating PMMA with UV light. A change in the dielectric properties of the encapsulant might influence the measured electroluminescence of our devices.

By comparing the simulated EL response from the pristine compounds to the experimental electroluminescence spectrum of the mixed layer of TCTA and TPBi (cf. Fig. 2b), a clear red shift can be observed for the mixed layer. This indicates that the EL originates from the D-A-exciplex (EX) species in the mixed film and occurs at $(2.70 \pm 0.01)$ eV, which is in agreement with previously reported values^28,46,47^. A similar EL response can be observed for all devices fabricated in this work. We can thus conclude that a good intermixture occurs at the interface between TCTA and TPBi, leading to an efficient exciplex formation. Interestingly, for the device E2-ACRIFIX$\circledR$ a significant difference in the CIE chromatic diagram^48^ was observed (see Fig. 2c and d)^49^. Additionally, the optoelectronic properties, especially the EQE (cf. Fig. 3b), and the magnetic field effect response are significantly lower, which can directly be attributed to a less efficient exciplex formation. We further extracted the dark-CELIV (charge extraction by linearly increasing voltage) mobility for all four devices. All of them exhibit mobilities in the $10^{-6} \, \text {cm}^2/\text {Vs}$ regime (cf. Fig. S2), which is in line with reported values for TADF materials^50–52^. However, significantly lower mobility was achieved for the E2-ACRIFIX$\circledR$ sample, which correlates with the results obtained by optoelectronic measurements and will be discussed further below. Additionally, impedance spectroscopy was carried out to extract the geometric capacitance, series, and parallel resistances by an adequate equivalent circuit model. The used model is depicted in Fig. S2-inset. For all devices, the values are similar and can be found in Fig. S2, respectively. The devices exhibit relatively low series resistance, which is attributed to a good charge carrier transport (cf. CELIV mobility under Fig. S2a). The parallel resistance lies, as expected, in the range of M$\Omega$, indicating a low leakage current. The capacitance, while demonstrating values within an expected range, is primarily influenced by the geometry of the electrodes and the properties of the organic materials. Conclusively, high-quality devices were fabricated despite the chosen simple layer stack structure and independently of the deposition method. Noticeably, the full solution processing seems to improve the formation of exciplexes and makes RISC more efficient. Nevertheless, a significant efficiency roll-off was detected, which most likely originates from non-radiative triplet processes. To determine the main loss mechanism, responsible for the efficiency roll-off, magnetic field effect measurements were carried out.

### Magnetic field effects in TCTA:TPBi exciplex devices

**Fig. 4:**
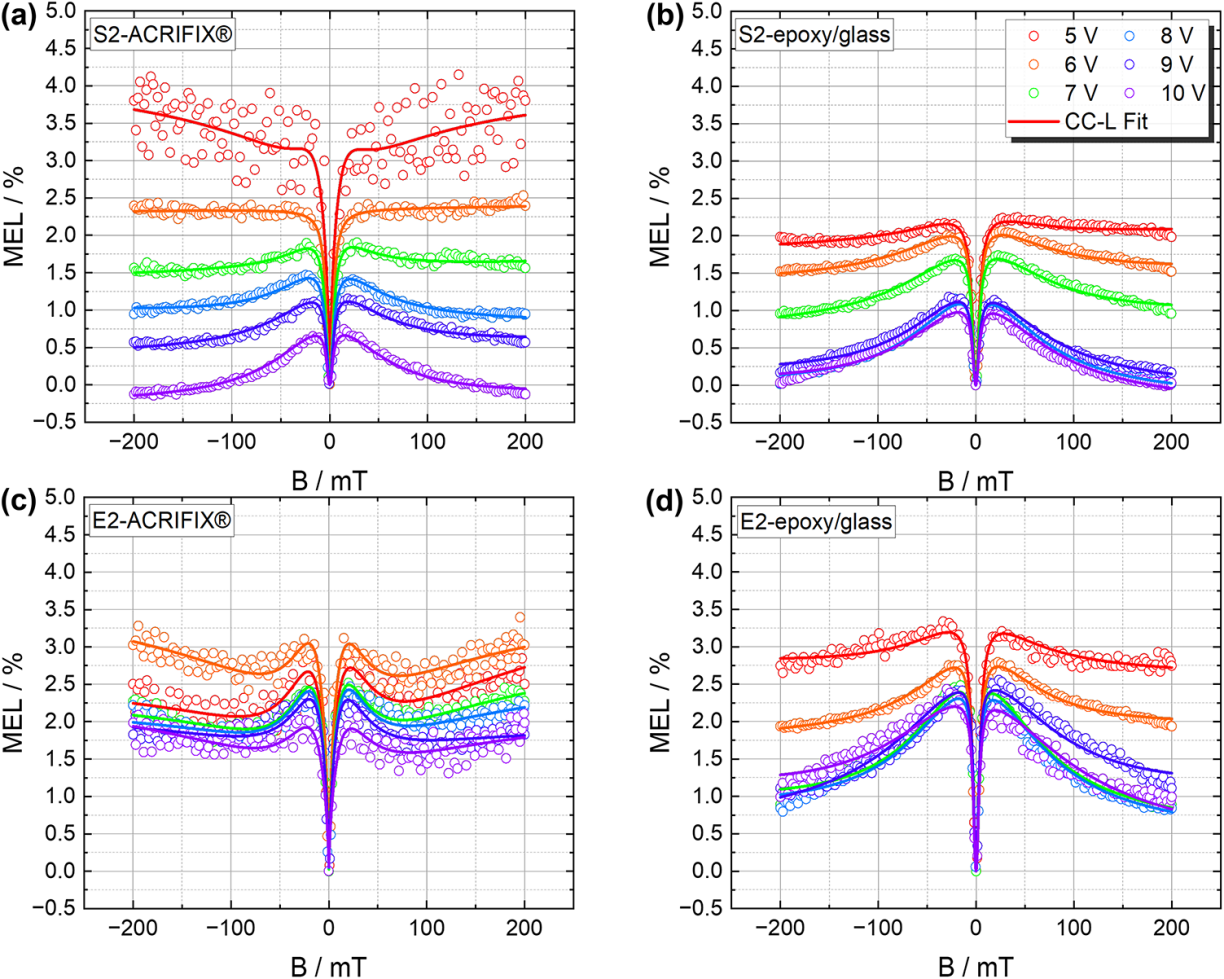
MEL response at bias voltages from ${5}\,{\hbox {V}}$ to ${10}\,{\hbox {V}}$, respectively for devices (**a**) S2-ACRIFIX$\circledR$, (**b**) S2-epoxy/glass, (**c**) E2-ACRIFIX$\circledR$, (**d**) E2-epoxy/glass. All diagrams depict the raw data points as dots (only every 20th measurement point is shown here) and the respective CC-L fit as solid lines. A strong contribution at magnetic fields exceeding $|20 |$ mT can be observed and is attributed to second-order processes.

Figure 4 presents the MEL response of the devices examined in this study at bias voltages ranging from $$5$$ to $10\,\mathrm{V}$. In most of the measured curves (represented by symbols in Fig. 4) we observed a positive MEL in the range of a few percent, which is in line with the predicted dependence of the MEL strength on the $$\mathrm {{HOMO}_\text {donor}}$$ − $$\mathrm {{LUMO}_\text {acceptor}}$$ gap for exciplex TADF materials^26^. If low magnetic fields are applied, the MEL value increases, and it decreases when the applied field exceeds $$|20 |$$ mT. As already mentioned in the introduction, the magnetic field effects in organic semiconducting materials have previously been attributed to PP interactions, originating from hyperfine interaction and the $$\Delta g$$ mechanism or to triplet processes^18^. It would, therefore, be desirable to relate the lineshape of the MEL to the responsible mechanisms. Recently, it was reported that MFE can be used to non-destructively determine the underlying mechanisms responsible for device degradation by the usage of the Cole–Cole fitting function^21–23,53^, if the charge carrier transport is dominated by dispersive dynamics of the polaron pairs, as it might be the case in an organic semiconductor such as the TADF layer used in this work. The Cole–Cole function can be found in Eq. (1).

1 $$\begin{aligned} \text {MEL}_\text {CC}(B) = {{MFE}_\text{LF} \cdot \left[ \frac{{1 + \left( \frac{B}{B_\text {LF}}\right) ^\alpha \cos \left( \frac{\pi \alpha }{2}\right) }}{1 + 2\left( \frac{B}{B_\text {LF}}\right) ^\alpha \cos \left( \frac{\pi \alpha }{2}\right) + \left( \frac{B}{B_\text {LF}}\right) ^{2\alpha }} - 1 \right] } \end{aligned}$$

with the half-width at half maximum (HWHM) $B_\text {LF}$, the dispersive parameter $\alpha$, and the maximum magnetic field effect $\mathrm {MFE_{LF}}$. Before an external magnetic field is applied, spin mixing between singlet and triplet states occurs. If an external magnetic field is applied, the spins of the polaron pairs align accordingly, and spin mixing is suppressed, leading to a change in the conductivity and hence in electroluminescence^18^. In most models, one assumes a well-defined lifetime value $\tau$ for the PP. However, in a disordered semiconductor system, such as the TADF emitters used in this work, the assumption of a distribution of the PP lifetime is more appropriate. Mondal et al.^22^ suggested that this dispersion in the PP lifetime might dominate the magnetic field effect response. This would be the consequence of the dephasing of the Larmor frequencies of electrons and holes originating from slightly different chemical environments, namely by the $\Delta g$ mechanism^18,22^. However, using the Cole–Cole function alone (Eq. (1)), it was not possible to fit our data (cf. Fig. S3).

This can be attributed to the fact that the Cole–Cole function is not sensitive with respect to strong high-field effect contributions. As shown in Fig. 4, an additional effect becomes significant at magnetic fields exceeding $|20 |$ mT, as no saturation of the MEL curves at higher magnetic field can be observed. Here, it is important to mention that all single-term models (Lorentzian, Non-Lorentzian, Cole–Cole) are not suited to describe strong HFE (see Fig. S3), originating from second-order processes^6,54^. We therefore extended the function in Eq. (1) by a Lorentzian term responsible for fitting the high-field part of the curves. The respective fitting function can be found in Eq. (2), with two additional parameters: the full width at half maximum (FWHM) $B_\text {HF}$ and the HFE ${MFE_\text{HF}}$ at $200\,\text {mT}$.

2 $$\begin{aligned} \text {MEL}_\text {CC-L}(B) = {{MFE}_\text{LF} \cdot \left[ \frac{{1 + \left( \frac{B}{B_{0_\text {LF}}}\right) ^\alpha \cos \left( \frac{\pi \alpha }{2}\right) }}{1 + 2\left( \frac{B}{B_{0_\text {LF}}}\right) ^\alpha \cos \left( \frac{\pi \alpha }{2}\right) + \left( \frac{B}{B_{0_\text {LF}}}\right) ^{2\alpha }} - 1 \right] } + {{MFE}_\text{HF} \cdot \left( \frac{B^2}{B^2 + {B_{0_\text {HF}}^2}} \right) } \end{aligned}$$

This model allows to separate the contribution of LFE and HFE, similar to the double-term models employed in earlier studies^6,18^. Additionally, it enables the determination of crucial parameters, such as the polaron pair lifetime distribution. Furthermore, it provides the ability to gain information about the disorder and degradation of the device. The absence of saturation can be attributed to the contribution from HFE, likely due to TPI or TTA effects. As triplet-excitons collide with charge carriers (polarons, p) (TPI), trion states are formed, where either a spin doublet or quartet is generated ($\mathrm {T_1 + p \rightarrow p^{\prime} + S_0}$)^55^. Note that the doublet is spin allowed, and hence the $\mathrm {EX_1}$ state contributing to the RISC can be quenched immediately via the energy transfer to the polaron. This process generates a “hot” polaron, which is high in energy and can dissociate chemical bonds. Consequently, these processes are responsible for the formation of charge traps and the decomposition of the material^20^. Since the dispersive parameter $\alpha$, related to the device degradation, does not change with increasing bias voltage, we assume that doublet intermediate states and the generation of hot polarons do not occur for the S2 and E2-epoxy/glass combination (cf. Figs. S4 and S5). Interestingly, for the device E2-ACRIFIX$\circledR$, a much lower $\alpha$ value was detected, implying the appearance of decomposed material or the dissociation of chemical bonds (cf. Fig. 6). Hence, the lower performance observed in those measurements can be attributed to loss mechanisms associated with TPI^20,55^. The observed line shape also matches previously reported fingerprint curves for this mechanism (cf. ref.^55^). We expect that quartet-trion intermediate states only play a minor role and are thus not further discussed. Since the curves observed for the other three devices (cf. Fig. 4) display a different line shape, another triplet mechanism has to be considered. The TTA process can be described as $\mathrm {T_1 + T_1 \rightarrow S_1 + S_0}$ and is also affected by the external magnetic field. Since the zero-field splitting (ZFS) exceeds the external magnetic field in the LFE regime^55^, the MEL response is positive, and the absolute MEL response increases with the magnetic field. However, in the HFE regime, the Zeeman splitting exceeds the ZFS, causing the MEL response to decrease. This characteristic fingerprint curve is observed in the S2 epoxy/glass and ACRIFIX$\circledR$ devices and in the E2 epoxy/glass sample. Interestingly, this effect does not dominate under low bias voltage^20^. To understand the decrease of the absolute MEL response by increasing bias voltage, we suggest a dependence on the operation conditions and device thickness as has been explained by Bergeson et al.^56^. Thereby, the recombination mobility is either (1) inversely proportional to the square of or (2) linearly dependent on the current density. The crossover regime is determined by the critical recombination mobility $\mathrm {\mu _c}$, which is simultaneously the point where the maximum magnetic field effect is expected. As can be seen in Fig. 4 for all devices the ${MFE_\text{max}}$ occurs at low bias voltage, wherefore we expect the operation to start at the critical recombination mobility (in between scenario (1) and (2)). By increasing bias voltage the MEL decreases as well as the OMC (cf. Fig. S6). For the lower bias voltages, the changes for OMC are negligible, wherefore the MEL constantly decreases. We attribute the effect to the switching from the intermediate scenario between (1) and (2) to scenario (1) where the current density depends non-linearly on the recombination mobility. Especially for the lower bias voltages, an alternation between the regimes leads to unstable results for the fit parameters (cf. Fig. S4a).

**Fig. 5:**
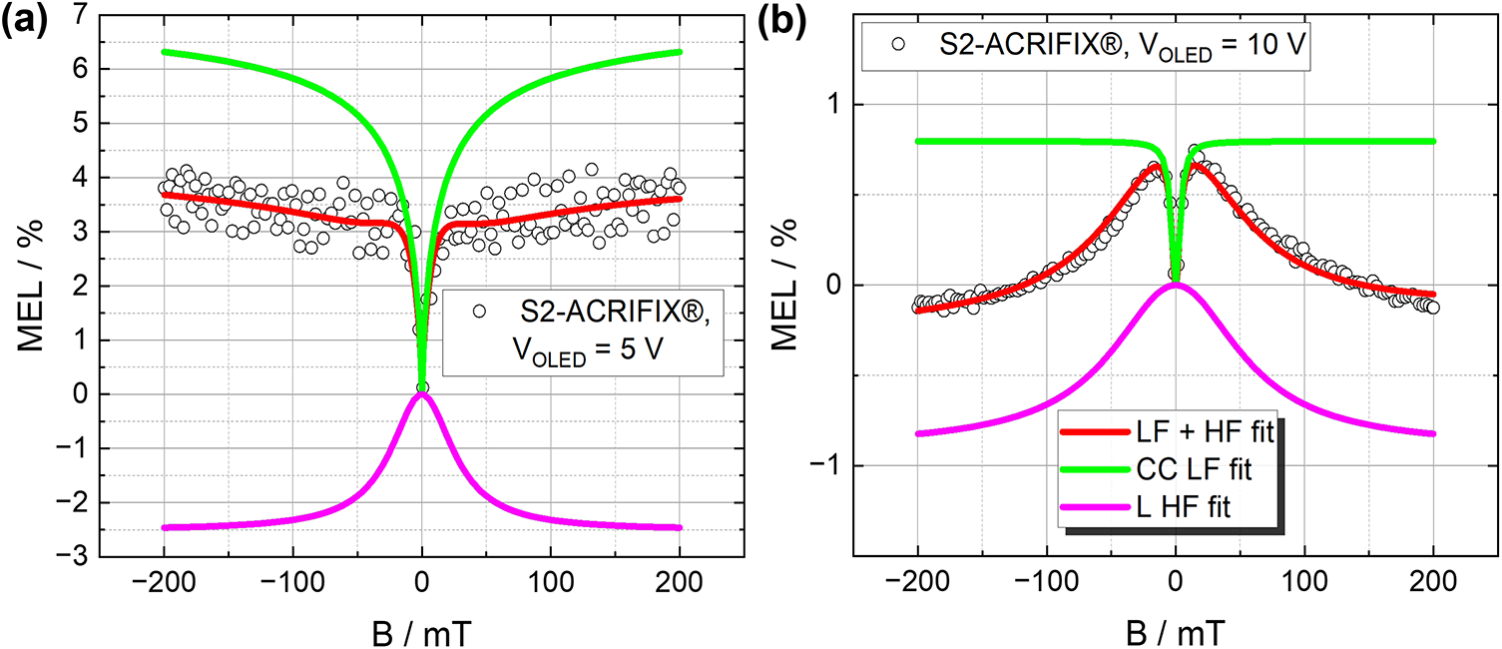
Fitting contributions of LFE and HFE from Eq. 2 exemplarily depicted for (**a**) S2-ACRIFIX$\circledR$ at $\mathrm {V_{OLED}}$ = 5   $\textrm{V}$ and (**b**) S2-ACRIFIX$\circledR$ at $\mathrm {V_{OLED}}$ = 10   $\textrm{V}$. The LFE is marked in green and the HFE in purple. The raw measurement data are plotted as dots and the combined fit as red line.

To unravel the loss mechanism causing the strong efficiency roll-off, the mean PP lifetime was calculated by $\tau _0 = \frac{\hbar }{\mu _{B} \cdot \Delta g \cdot B_{0_\text {LF}}}$^21^, with the constant values for $\Delta \,g$ = 0.002, the Planck constant $\hbar$ and the Bohr magneton $\mu _{B}$, from the data shown in Fig. 4. By extracting the values $\alpha , \tau _0$ and $B_{0_\text {LF}}$ it was found that the devices E2-epoxy/glass and S2-epoxy/glass as well as S2-ACRIFIX$\circledR$ exhibit high dispersive parameters $\alpha$, partly restoring the Lorentzian behavior (for $\alpha = 1$). Since the spin mixing in polaron pairs is influenced not only by hyperfine interactions but also by the difference in *g*-values between the electron and hole forming the PP, a strong influence of this effect would cause a lower $\alpha$ value. Since this is not the case for these devices, the LFE alone cannot account for the substantial efficiency roll-off observed in the EQE measurements. To provide a clearer understanding of the underlying LFE and HFE mechanisms, these contributions were separately plotted in Fig. 5 for S2-ACRIFIX$\circledR$ at $5\,\textrm{V}$ and $10\,\textrm{V}$, respectively. A negative LFE contribution would indicate a dominating RISC effect^15,17^, while a positive contribution originates from hyperfine induced ISC or $\Delta g$ induced RISC^55^.

**Fig. 6:**
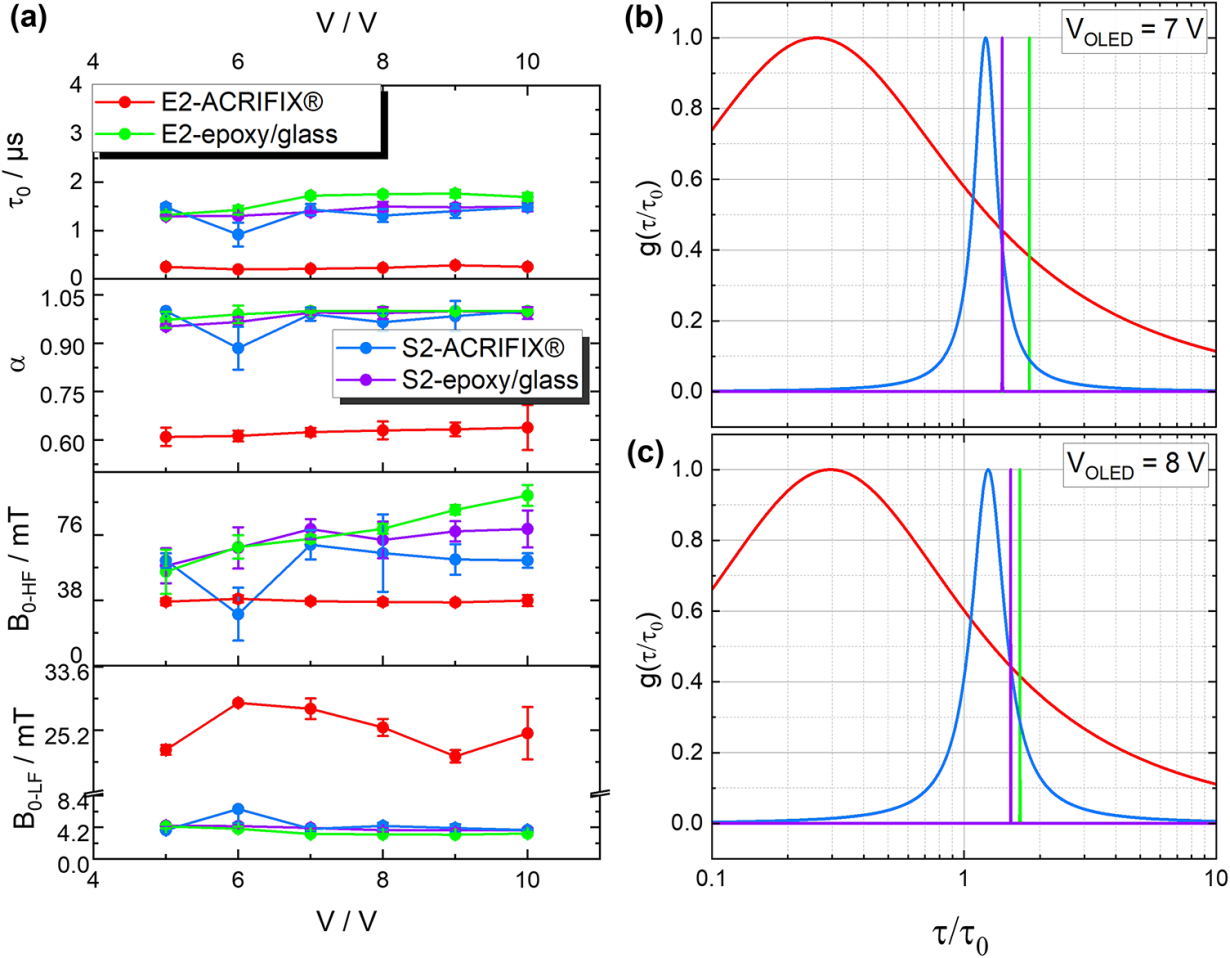
(**a**) Mean values for $B_{0_\text {LF}}$, $B_{0_\text {HF}}$, $\alpha$ and the mean PP lifetime $\tau _0$ (from bottom to top) with the standard deviation extracted from ten separate measurements, (**b**) and (**c**) the PP lifetime distribution $\text {g}(\tau )$ for the four different samples at a bias voltage of 7 V and 8 V.

By increasing the bias voltage, the broadening of the curve decreases, cf. $B_{0_\text {LF}}$ (cf. Fig. 6a). Simultaneously, the HFE contribution broadens (cf. Fig. 6a—$B_{0_\text {HF}}$ and Fig. 5). This phenomenon increases with increased bias voltage, reflecting a decrease of the exciton lifetime^15,57,58^. Considering the percentage share of the total effect at $\mathrm {V_{OLED}}=5\,\mathrm{V}$, the LFE contributes significantly with $74\%$, while the HFE accounts for only $26\%$. However, at a higher bias of $\mathrm {V_{OLED}}$ = $10\,\mathrm{V}$, the HFE contribution increases dramatically to 51%, becoming the dominant mechanism over the LFE. This shift in mechanism dominance aligns with the anticipated characteristics of TTA.

### Distribution of the polaron pair lifetime

As previously reported in ref.^22^, in addition to the mean PP lifetime $\tau _0$, the distribution of the PP lifetime can be described by the following formula Eq. (3), where $\alpha$ and $B_{0_\text {LF}}$ were taken from the Cole–Cole part of the fitting function of Eq. (2).

3 $$\begin{aligned} \begin{aligned} \text {g}(\tau ) = \frac{1}{2 \pi } \cdot \frac{\sin (\pi \alpha )}{\cosh (\alpha \ln (\frac{\tau }{\tau _0})) + \cos (\pi \alpha )} \end{aligned} \end{aligned}$$

$\tau _0$ can be determined as described above. Figure 6b,c exemplarily presents the results for all devices at bias voltages of 7 V and 8 V. For the devices encapsulated by epoxy/glass a $\delta$-distribution was obtained, which is correlated to the observed restored Lorentzian behavior of the MEL lineshape in the low-field regime. So within the measurement time, no degradation behavior was observed. Notably, as the bias voltage increases, the contribution of the low-field effect (LFE) becomes narrower (cf. Fig. 5). This behavior was already observed in previous studies on $\text {Alq}_3$ devices. We attribute this behavior to the filling of trap states, as explained by the drift-diffusion model. Consequently, while the recombination dynamics change, the current flow increases. This effect is further evidenced by the switch of the OMC sign (cf. Fig. S6). For the E2-ACRIFIX$\circledR$ device, significantly lower mean polaron pair (PP) lifetimes were observed, along with strong charge carrier scattering contributions^17,20^, as indicated by the broader LFE contributions compared to its epoxy/glass-encapsulated counterpart. Additionally, a broad distribution of PP lifetimes was detected in the E2-ACRIFIX$\circledR$ device, consistent with other measurements from this study. Combined with the low dispersive parameter $\alpha$ and the significantly reduced device performance (see Figs. S2 and 3b), these findings suggest that the combination of liquid processing and vapor deposition with ACRIFIX$\circledR$ encapsulation leads to lower device quality as compared to the other processing methods. On the contrary, S2-ACRIFIX$\circledR$ exhibits a high mean PP lifetime with a narrow distribution, which makes this material system highly suitable for further studies in the field of gas sensing. We recently published a perspective using OLED-based structures as gas sensors for hydrogen detection in fuel cell systems^29^. This approach leverages the fact that an increase in hydrogen atoms is expected to alter the hyperfine interactions (cf. ref.^59^). As a result, a stable, semipermeable encapsulation layer is essential to ensure reliable sensor performance. The unintentional doping in combination with the solution processing resulted in good performance of the device. The mean PP lifetimes for all devices are displayed in Table 2 exhibiting values in the range of  1.5 µs, except for E2-ACRIFIX$\circledR$. This device exhibits a mean PP lifetime significantly lower $~ (0.23 \pm 0.05)$ µs, in agreement with the already discussed performance of this device. Similar values for the PP lifetime, as determined for the other devices, have been observed before for the small molecule $\mathrm {Alq_3}$ resulting in a similar $\textrm{MFE}$ strength^22^.

**Table 2:** Mean PP lifetime for all samples at bias voltages of 7 V and 8 V, respectively.

Sample	Mean PP lifetime (μs)	Bias voltage (V)
S2-ACRIFIX$\circledR$	$1.44 \pm 0.11$	7
S2-ACRIFIX$\circledR$	$1.31 \pm 0.13$	8
S2-epoxy/glass	$1.38 \pm 0.02$	7
S2-epoxy/glass	$1.50 \pm 0.10$	8
E2-ACRIFIX$\circledR$	$0.22 \pm 0.05$	7
E2-ACRIFIX$\circledR$	$0.24 \pm 0.06$	8
E2-epoxy/glass	$1.72 \pm 0.06$	7
E2-epoxy/glass	$1.75 \pm 0.05$	8

### Assessment of the model quality using k-fold cross validation

**Fig. 7:**
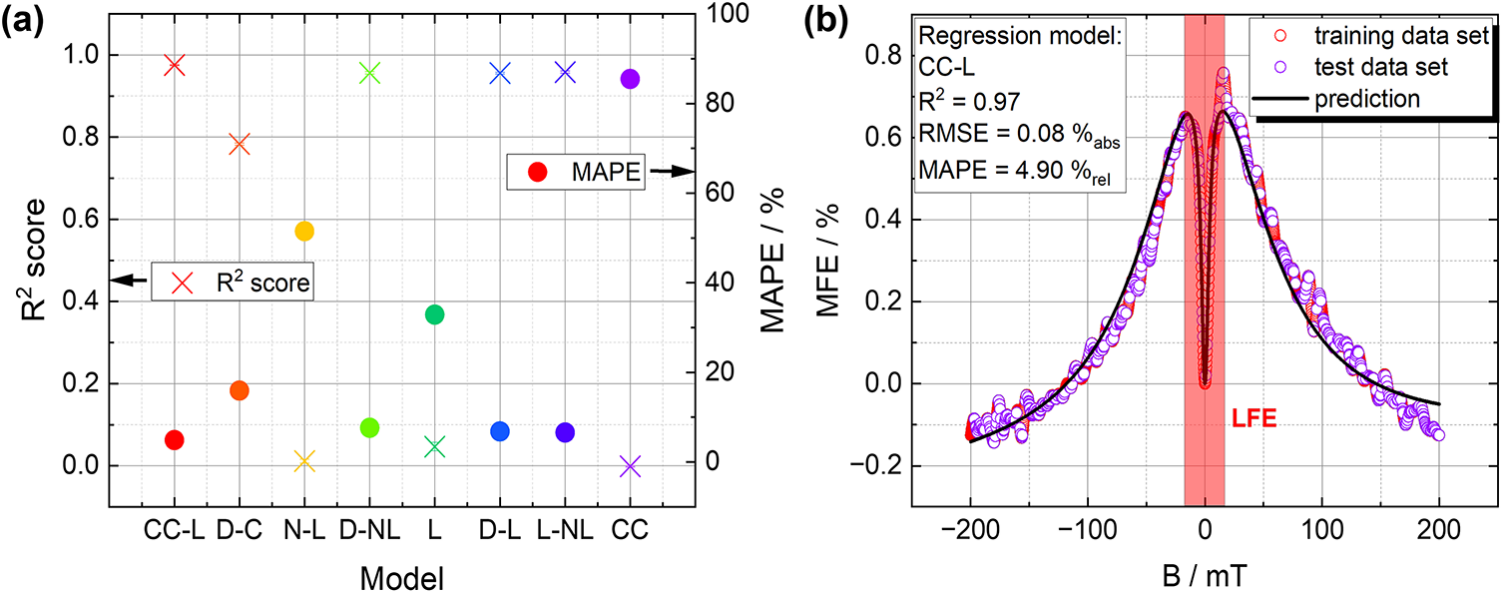
(**a**) $R^2$ score and the *MAPE* evaluated by fitting the for several models, (**b**) the predicted value upon the best model, extracted from 5-fold cross-validation, namely the Cole–Cole-Lorentzian model, showing the original data for S2-ACRIFIX-$10 \textrm{V}$, the training data set (80%), and the test data set (20%).

To evaluate the quality of the new fitting function, we first applied k-fold cross-validation (k = 5)^60^ to $80\%$ of the randomly selected training data set based on the measured data (cf. Fig. 7b). This technique can ensure the generalization capability. We compared the suitability of different Lorentzian, non-Lorentzian, and combined regression models, (cf. ref.^18,20^) to probe the estimator performance. Detailed information regarding the applied functions and their abbreviations can be found in the SI Table S2. For comparison, the $R^2$ score and the Mean Average Percentage Error (*MAPE*) were calculated and are depicted in Fig. 7a for all used regression functions. The formula for the $R^2$ score reads in Eq. (4) and for the *MAPE* in Eq. (5), respectively.

4 $$\begin{aligned} R^2= & 1 - \frac{\sum _{i=1}^{n} (y_i - {\hat{y}}_i)^2}{\sum _{i=1}^{n} (y_i - {\bar{y}})^2}, \end{aligned}$$

5 $$\begin{aligned} MAPE= & \frac{1}{n} \sum _{i=1}^{n} \left| \frac{y_i - {\hat{y}}_i}{y_i} \right| \cdot 100, \end{aligned}$$

with $y_i =$ actual value of the target variable, ${\hat{y}}_i =$ predicted value from the model, ${\bar{y}} =$ mean of the actual value $y_i$ and the number of observations *n*^61^. Please note that the $R^2$ score can also take on negative values, indicating that the model performs worse than a simple mean prediction, as it fails to capture the variability in the data adequately. As can be seen, the single-term models, namely Lorentzian, non-Lorentzian, and Cole–Cole performed the worst, with a significant *MAPE* in the range between 30 and $80\%_\text {rel}$. It should be noted that the *MAPE* represents the relative error in percentage (relative, “rel”), while the Root Mean Squared Error (*RMSE*) reflects the absolute error. Since the original unit of measurement data is percentage, the *RMSE* is also expressed in percentage, specifically as an absolute error (“abs”)(cf. Fig. 7b). The $R^2$ score, indicating how well the predictor variable accounts for the variation in the response variable, was approximately zero for these models, suggesting that they are highly unsuitable for fitting the measured data. For the double-term models, namely Double-Non-Lorentzian, Double-Lorentzian, Lorentzian-Non-Lorentzian as well as the Cole–Cole-Lorentzian model, the ${R^2}$ score consistently exceeded $95\%$, with the Cole–Cole-Lorentzian model achieving the highest score and the lowest *MAPE*. The Double-Cole–Cole model revealed in the lowest ${R^2}$ score for the double-term models, implicating, that the Cole–Cole function is highly unsuitable to fit the HFE. This aligns with our assumptions, as the theory for the Cole–Cole function is based on the $\Delta$g difference, which can only be detected in the LFE regime^22^. Thus, the best suitable model, namely Cole–Cole-Lorentzian function, was applied to the $20\%$ test data set, revealing a *RMSE* of $0.08\%_\text {abs}$, an ${R^2}$ of 0.97 and a *MAPE* of $4.90\%_\text {rel}$. Consequently, it was demonstrated that the application of this model resulted in neither overfitting nor underfitting, accurately replicating the MEL response (cf. Fig. 7b).

## Conclusion

In this work, we investigated the magnetic field effects in third generation blue OLEDs fabricated by various combinations of vapour phase and liquid phase processing. We demonstrated a universal approach using a Cole–Cole-Lorentzian fitting function to approximate the MFE response, which is highly suitable for unraveling processes involving triplet excited states and dispersive dynamics of polaron pairs. Consequently, the investigation of MFEs can contribute to the elucidation of PP dynamics. Additionally, this method enables non-destructive identification of the dominant second-order mechanism, which is primarily observed in the HFE of the material system. Optoelectrical investigations confirmed the device’s stability, while DFT calculations showed that the singlet-triplet gap for the TCTA:TPBi material combination is minimal, approaching zero, and thus easily accessible at room temperature. These findings support the observations from EL investigations, where a significant red shift was seen for the material combination compared to the individual components. For this material system, the primary loss mechanisms were identified as triplet processes, namely TTA and TPI. Consequently, the RISC process becomes less effective at higher bias voltages, leading to a reduction in the absolute MEL effect strength due to the increased influence of second-order processes. This reduction is reflected in the broadening of the HFE response, while a marked shift in the percentage share of the total effect from both LFE and HFE becomes apparent, with HFE contributions dominating at higher bias voltages. By considering the dispersive parameter $\alpha$, it is possible to distinguish between degraded and non-degraded devices, where a $\delta$-like distribution is expected for non-degraded devices, while degraded devices exhibit a lower $\mathrm {\tau _0}$ with a broader PP lifetime distribution. The LFE response is attributed to decreased scattering processes with increasing bias voltage, related to trap filling. Additionally, insights into the PP lifetime distribution can be gleaned from the LFE, revealing its dispersive nature that reduces the MEL. The high quality of the fitting function was proven through the use of the k-fold cross-validation algorithm. The highest $R^2$ score with 97% was evaluated by the application of the Cole–Cole-Lorentzian fit. The transferability was further investigated by the application of the function to several device structures, exhibiting various lineshapes. When comparing two encapsulants, potential doping effects were observed with the application of ACRIFIX$\circledR$, where the highest MEL response of $(3.67 \pm 0.53)\,\%$ by an $\mathrm {{EQE_{max}}}$ = $(8.62 \pm 1.99)\,\%$ for S2-ACRIFIX$\circledR$ was measured. A dispersive character of the PP lifetime was observed only with the ACRIFIX$\circledR$ encapsulation, suggesting the possibility of unintentional doping. Due to increased MFE by the usage of ACRIFIX$\circledR$, we propose it for further sensing applications, particularly in magnetic field sensing technology on flexible substrates with arbitrary geometry. In summary, this novel approach has implications for improving organic devices’ performance and exploring new applications in sensing technology.

## Methods

### Materials

As substrates, we used pre patterned ITO glass substrates provided by Ossila. Poly-(3,4-ethylendioxythiophene)-poly-(styrene sulfonate) (PEDOT:PSS) from Heraeus (Clevios™-PEDOT:PSS). Additionally, Molybdenum(VI) oxide powder ($\mathrm {MoO_3}$), poly(9-vinylcarbazole) (PVK), and lithium fluoride (LiF) were purchased from Sigma-Aldrich. Tris(4-carbazoyl-9-ylphenyl)amine (TCTA) and 2,2′,2″-(1,3,5-benzinetriyl)-tris(1-phenyl-1-H-benzimidazole) (TPBi) were obtained from Ossila. All materials were used without further purification.^28^. The PEDOT:PSS solution was diluted with isopropanol (IPA) at a ratio of 1:0.04 w%. $\mathrm {MoO_3}$ was mixed with PEDOT:PSS at a ratio of 0.02:1. Prior to this, the $\mathrm {MoO_3}$ powder was dissolved in ammonium hydroxide ($\mathrm {NH_3OH}$) ($0.25\,\mathrm {g/ml}$). The initial solutions for TCTA and PVK were prepared to be $15\,\mathrm {mg/ml}$ and $10\,\mathrm {mg/ml}$, respectively. The two solutions were mixed afterward at a mass ratio of 1.5:1. TPBi was dissolved at a ratio of $1.5 \ \mathrm {mg/ml}$ IPA. Prior to device fabrication, all solutions were treated in an ultrasonic bath at $80 \ ^\circ \textrm{C}$ for at least 30 min and filtered by a nylon syringe with a pore size of $0.22 \ \mathrm {\upmu m}$. The remaining materials were deposited via thermal evaporation.

### Experimental details

The TADF devices were fabricated with the following structure (cf. 1: ITO (115 nm, purchased by Ossila, prepatterned anode (six pixels)/ PEDOT:PSS + $\mathrm {MoO_3}$ (35 nm)/ PVK:TCTA (1 : 1.5 ratio, $20\,\text{nm}$)/ TPBi ($20 \ \textrm{nm}$)/ LiF ($0.8\ \textrm{nm}$)/ Al ($110 \ \textrm{nm}$). Before the PEDOT:PSS + $\mathrm {MoO_3}$ was deposited, the samples were cleaned by alkalic acid + DI water and IPA in an ultrasonic bath for 30 min, respectively. Afterwards, the samples were dried by nitrogen. The surface was further activated by a UV-Ozon treatment with a self-made setup (after ref.^62^) for 35 min. TCTA was spin coated under nitrogen atmosphere at 3000 rpm, (acceleration $300 \ \mathrm {rpm/s}$) for 30 s and post annealed at 130 $^\circ$C for 30 min. TPBi was either evaporated (E2) at a constant rate of 1  Å/s or spin coated (S2) at $2000 \ \textrm{rpm}$ (acceletarion $300\,\mathrm {rpm/s}$) for 30 s and post annealed at 130 $^\circ$C. LiF and Al were evaporated at a constant rate of 0.1  Å/s and 5  Å/s, respectively. The deposition via thermal evaporation in the vacuum chamber was performed at a pressure of $10^{-6} \ \textrm{mbar}$. For more details we refer to our previous work^28^ (cf. Materials and Methods and Figure 2.1.3 (Device E2 and S2)). The devices were encapsulated either by epoxy resin and a glass slide or by ACRIFIX$\circledR$ (based on polymethylmethacrylat (PMMA) dissolved in dichloromethane). Both encapsulations were post treated by a UV lamp for 15 minutes. All measurements were performed at room temperature, where the external magnetic field was applied out of plane to the sample. A constant bias voltage was applied to the sample by using a Keithley 2636A source measure unit. The current was detected by a self-developed electrical set up, including a trans-impedance amplifier. Additionally, the electroluminescence response was detected with a photodiode, and its signal was amplified using a low-noise current preamplifier from Stanford Research Systems (SR570). Both signals were detected using an ADC module (24 bit ADC ADS1256 from Texas Instruments in an 11010 high-precision AD/DA board from waveshare) as well as an ESP-32 microcontroller with a sample rate of $866\,\text{Hz}$. A triangular voltage waveform (amplitude = 10 V, duty cycle = $50\%$, frequency = 0.1 Hz) was applied to the bipolar power supply (KEPCO-$100 \ \textrm{W}$). This power supply was connected to the magnet, which swept the magnetic field back and forth. Consequently, the measurement time for each ramp was 5 seconds. The PL spectra used for the simulation of the EL spectra in Fig. 2b was recorded by a Cary Fluorescence spectrometer, where the excitation wavelength was chosen to be $\mathrm {\lambda _{ex}}$ = $330 \ \textrm{nm}$. The UV-vis absorption spectra were measured using a Cary 60 UV-vis spectrometer. The baseline was determined using quartz glass and subtracted from the spectra of interest. To perform photometric characterization of the fabricated OLEDs, a Gigahertz-Optik UMBB-210 integrating sphere (sphere diameter of $210 \ \textrm{mm}$) was utilized in conjunction with a calibrated photodiode PD-1101 (measuring range from $250\,\text{nm}$ to $1100 \ \textrm{nm}$). The optical radiation dependence on wavelength was evaluated using an Ocean Optics Flame-S-UV-VIS-ES spectrometer. The OLED voltage supply and photodiode current measurement were performed using a Keysight B2902A source measure unit.

### Density functional theory calculations

DFT calculations were performed using the Orca 5.0 software^63^ using the O3LYP hybrid functional^40^. The functional was selected after testing several exchange-correlation functionals for their ability to reproduce the experimental HOMO and LUMO levels of TCTA. The geometrical counterpoise correction (gCP)^64^ and D4 dispersion correction^65,66^ were employed to treat basis set superposition errors and long range London dispersion, respectively. The Karlsruhe split valence basis set def2-SVP^67^ was used for coarse pre-optimization of the molecule geometries. The final optimizations and TDDFT calculations utilized the larger Karlsruhe triple zeta valence basis set def2-TZVP. For all calculations the RIJCOSX approximation was employed to speed up calculation time^68^. Possible dimer configurations of the two molecules were generated using the Conformer-Rotamer Ensemble Sampling Tool (CREST)^69^, which calculated the optimal structures at the extended tight binding level of theory (GFN2-xTB^70^). CREST found 15 additional unique dimer configurations within a $6\,\mathrm {kcal/mol}$ ($0.26 \ \textrm{eV}$) window of the lowest energy structure. Two of these had a calculated total energy less than $0.1 \ \mathrm {kcal/mol}$ ($4.3 \ \textrm{meV}$). All calculations on the dimers were performed for these two along with the lowest energy dimer configuration. The three dimers showed virtually identical HOMO-, LUMO-, singlet- and triplet levels with maximum differences of less than $0.01 \ \textrm{eV}$ in every case, well below the inherent inaccuracy of the DFT method.

## Supplementary Information

Supplementary Information.
