# Supplementary material for: Analysis of polaron pair lifetime dynamics and secondary processes in exciplex driven TADF OLEDs using organic magnetic field effects
Source: Sci Rep. 2024 Dec 17;14:30520. doi: 10.1038/s41598-024-82060-z (PMC11652625; doi:10.1038/s41598-024-82060-z)
Supplement: Supplementary file 1 — Supplementary Information. [file 41598_2024_82060_MOESM1_ESM.pdf]

Supplementary Information
Analysis of polaron pair lifetime dynamics and secondary processes in exciplex driven TADF OLEDs using organic magnetic field effects

Annika Morgenstern[1,*], Dominik Weber[2], Lukas Hertling[1,4], Konstantin Gabel[3], Ulrich T. Schwarz[3], Daniel Schondelmaier[2], Dietrich R. T. Zahn[1,4], and Georgeta Salvan[1,4*]

[1]Semiconductor Physics, Chemnitz University of Technology, Institute of Physics, Chemnitz, 09126, Germany
[2]Nanotechnology, University of Applied Sciences Zwickau, Physical Engineering and Computer Science, Zwickau, 08056, Germany
[3]Experimental Sensor Science, Chemnitz University of Technology, Institute of Physics, Chemnitz, 09126, Germany
[4]Center of Materials, Architectures and Integration of Nanomembranes (MAIN), Chemnitz University of Technology, Chemnitz, 09126, Germany
[*]salvan@physik.tu-chemnitz.de, annika.morgenstern@physik.tu-chemnitz.de

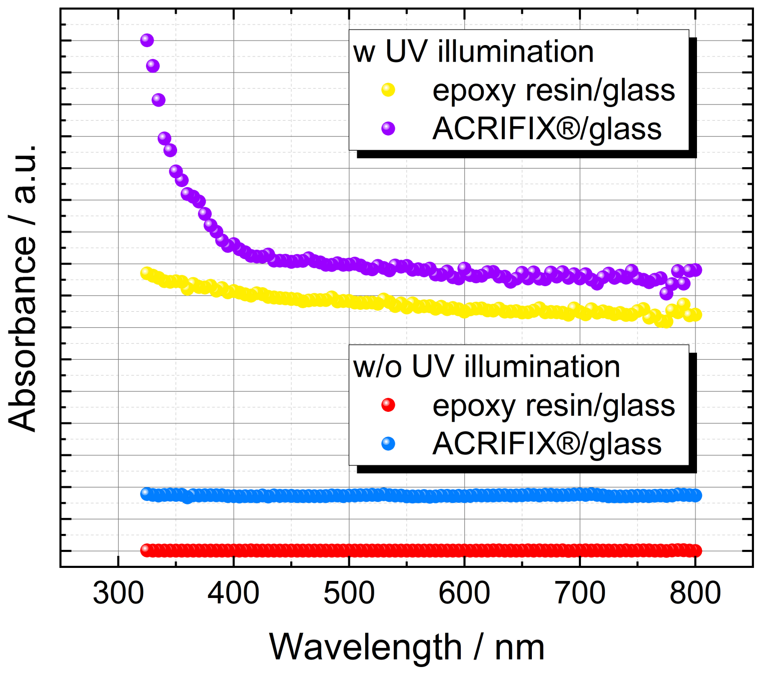

Figure S1. Absorption spectra obtained for epoxy resin and ACRIFIX® on glass substrates, respectively. The samples were once illuminated by UV light for 15 minutes (as done for all devices in this study) (w) and compared to samples that were not illuminated (w/o). The absorption onset is only visible for the ACRIFIX® when illuminated by UV light, increasing the possibility for unintentional doping upon illumination.

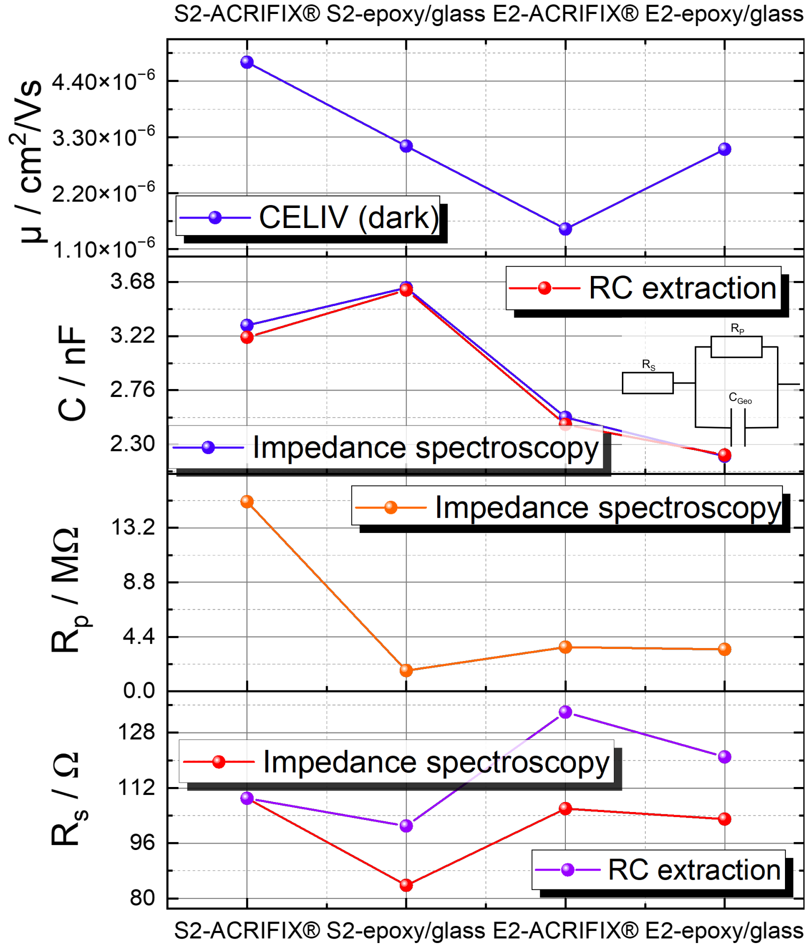

Figure S2. Extracted values for the (a) series and (b) parallel resistance as well as the (c) geometric capacitance from impedance spectroscopy obtained by the equivalent circuit model (see inset) depicted in panel three (from bottom to top), (d) charge carrier mobilities of the samples.

Table S1. Max. EQE determined from the diagram in Figure 3 (b).

**Sample**	**max. EQE / %**
S2-ACRIFIX®	8.62 ± 1.99
S2-epoxy/glass	2.42 ± 0.54
E2-ACRIFIX®	0.20 ± 0.76
E2-epoxy/glass	13.52 ± 3.07

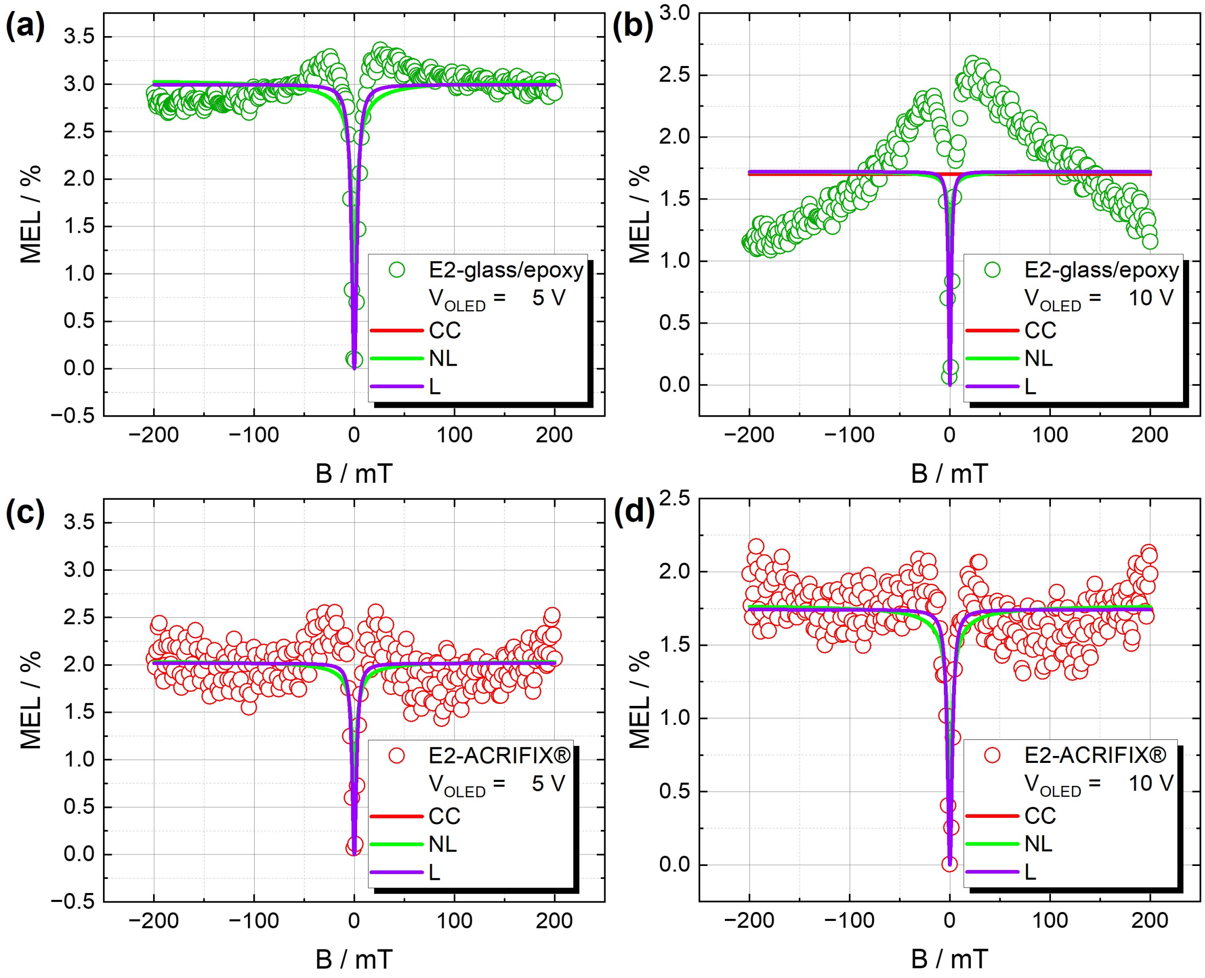

Figure S3. (a) Non-matching CC-, L- and NL-Fit for E2-epoxy/glass device at bias voltages of (a) 5 V and (b) 10 V, and E2-ACRIFIX device at bias voltages of (c) 5 V and (d) 10 V. As can be seen the fit is arbitrarily off and does not well reproduce the measured data. Especially for higher bias voltage a clear offset is observed. The CC- and L- fit are overlapping in most cases.

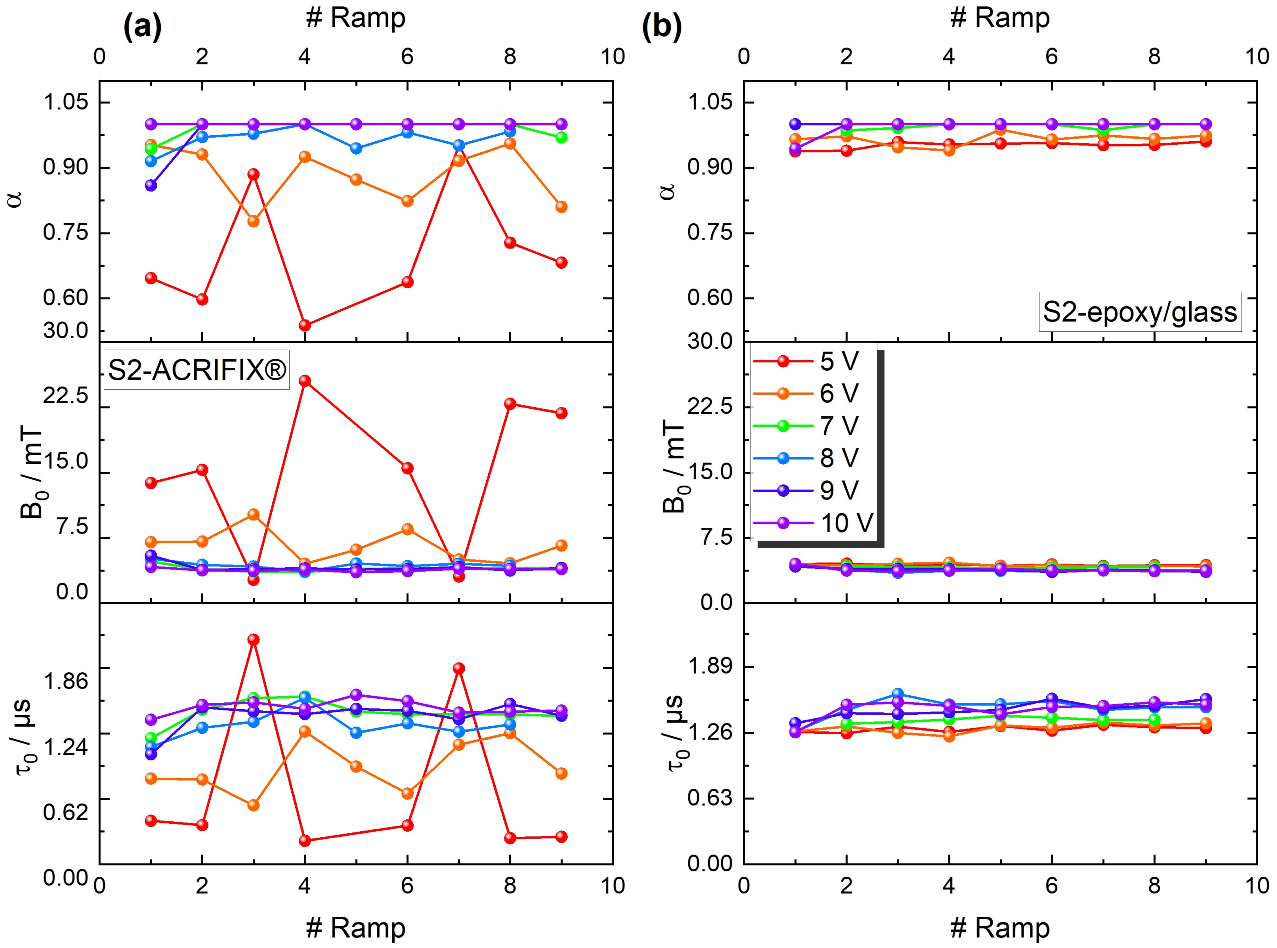

Figure S4. Results for the dispersive parameter α, the HWHM $B_{0_{\mathrm{LF}}}$ and the mean polaron pair lifetime τ_0 (from top to bottom) for (a) device S2-epoxy/glass and (b) S2-ACRIFIX®. The values were obtained from ten separate measurements (named ramp) and at different bias voltages.

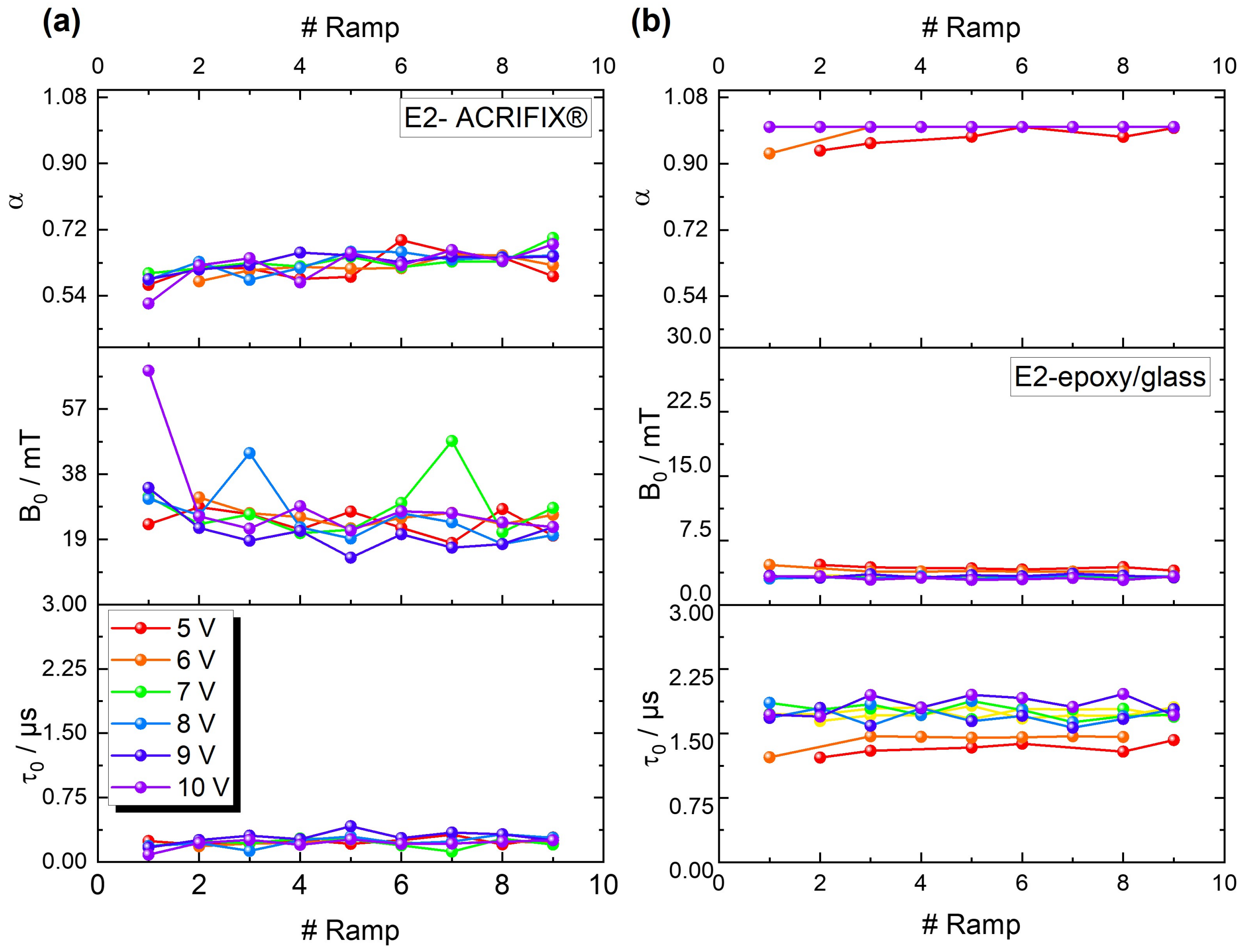

Figure S5. Results for the dispersive parameter α, the HWHM $B_{0_{\mathrm{LF}}}$ and the mean polaron pair lifetime τ_0 (from top to bottom) for (a) device E2-epoxy/glass, and (b) E2-ACRIFIX®. The values were obtained from ten separate measurements (named ramp) and at different bias voltages.

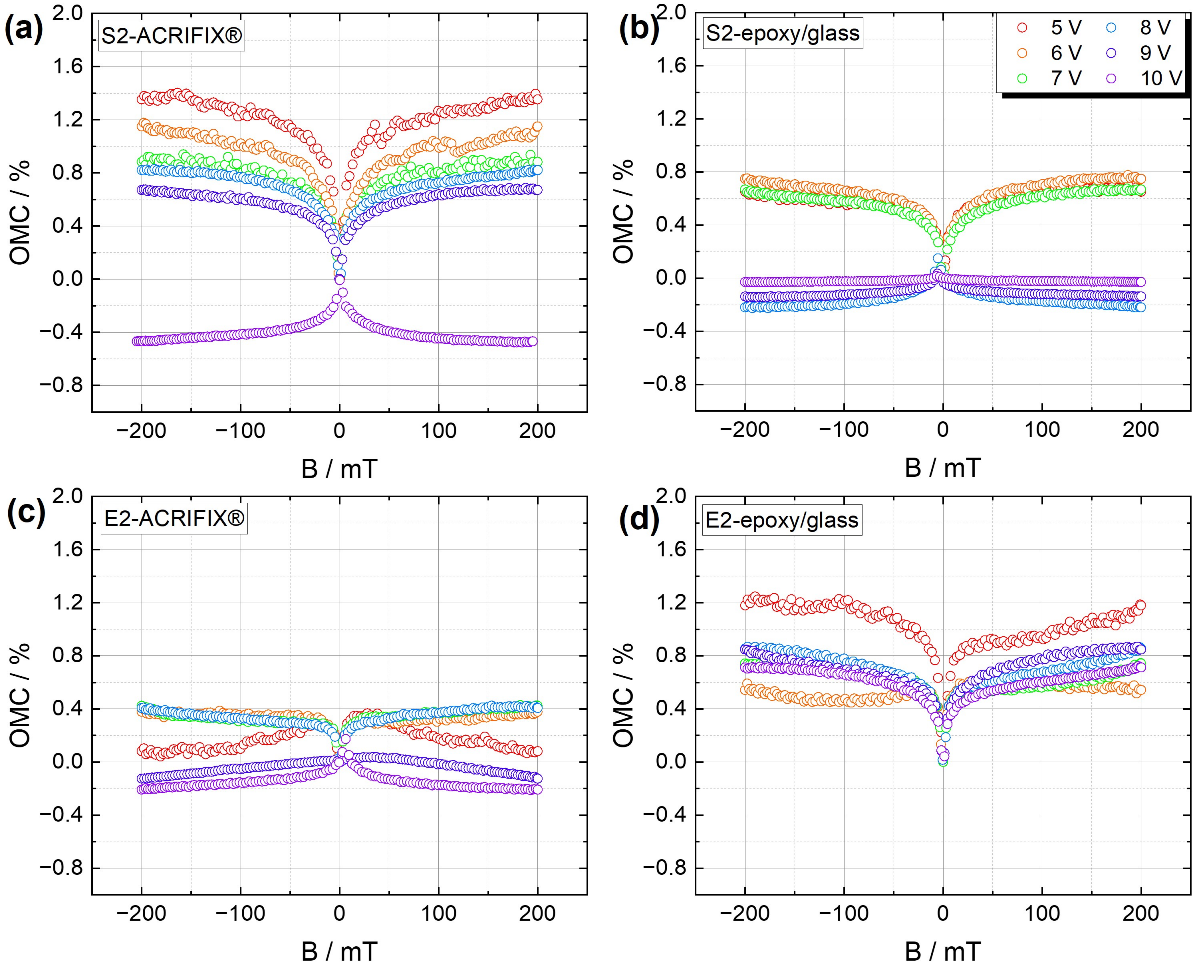

Figure S6. OMC response for devices (a) S2-ACRIFIX®, (b) S2-epoxy/glass, (c) E2-ACRIFIX®, and (d) E2-epoxy/glass. At higher bias voltage the OMC sign switches. As the main text explains, this can be attributed to exciton dissociation increasing the device current. The correlated MEL response does also show a stronger influence of the negative component at higher bias voltage.

Table S2. Fitting functions commonly used to fit magnetic field effects, which are used here for 5-fold cross-validation to determine the most suitable model for the measured data

Function name	Abbreviation	Formula
Cole-Cole	CC	$\mathrm{MEL}_{\mathrm{CC}}(B) = MFE_{\max} \cdot \left(\frac{1+\left(\frac{B}{B_0}\right)^{\alpha}\cos\left(\frac{\pi\alpha}{2}\right)}{1+2\left(\frac{B}{B_0}\right)^{\alpha}\cos\left(\frac{\pi\alpha}{2}\right)+\left(\frac{B}{B_0}\right)^{2\alpha}} - 1 \right)$
Lorentzian	L	$\mathrm{MEL}_{\mathrm{L}}(B) = MFE_{\max} \cdot \left(\frac{B^2}{B^2+B_0^2} \right)$
Non-Lorentzian	NL	$\mathrm{MEL}_{\mathrm{NL}}(B) = MFE_{\max} \cdot \left(\frac{B^2}{(B+B_0)^2} \right)$
Double-Cole-Cole	D-CC	$\mathrm{MEL}_{\mathrm{D\text{-}CC}}(B) = MFE_{\mathrm{LF}} \cdot \left(\frac{1+\left(\frac{B}{B_{\mathrm{LF}}}\right)^{\alpha}\cos\left(\frac{\pi\alpha}{2}\right)}{1+2\left(\frac{B}{B_{\mathrm{LF}}}\right)^{\alpha}\cos\left(\frac{\pi\alpha}{2}\right)+\left(\frac{B}{B_{\mathrm{LF}}}\right)^{2\alpha}} - 1 \right) + MFE_{\mathrm{HF}} \cdot \left(\frac{1+\left(\frac{B}{B_{\mathrm{HF}}}\right)^{\alpha}\cos\left(\frac{\pi\alpha}{2}\right)}{1+2\left(\frac{B}{B_{\mathrm{HF}}}\right)^{\alpha}\cos\left(\frac{\pi\alpha}{2}\right)+\left(\frac{B}{B_{\mathrm{HF}}}\right)^{2\alpha}} - 1 \right)$
Double-Lorentzian	D-L	$\mathrm{MEL}_{\mathrm{D\text{-}L}}(B) = MFE_{\mathrm{LF}} \cdot \left(\frac{B^2}{B^2+B_{\mathrm{LF}}^2} \right) + MFE_{\mathrm{HF}} \cdot \left(\frac{B^2}{B^2+B_{\mathrm{HF}}^2} \right)$
Double Non-Lorentzian	D-NL	$\mathrm{MEL}_{\mathrm{D\text{-}NL}}(B) = MFE_{\mathrm{LF}} \cdot \left(\frac{B^2}{(B+B_{\mathrm{LF}})^2} \right) + MFE_{\mathrm{HF}} \cdot \left(\frac{B^2}{(B+B_{\mathrm{HF}})^2} \right)$
Lorentzian-Non-Lorentzian	L-NL	$\mathrm{MEL}_{\mathrm{L\text{-}NL}}(B) = MFE_{\mathrm{LF}} \cdot \left(\frac{B^2}{B^2+B_{\mathrm{LF}}^2} \right) + MFE_{\mathrm{HF}} \cdot \left(\frac{B^2}{(B+B_{\mathrm{HF}})^2} \right)$
Cole-Cole-Lorentzian	CC-L	$\mathrm{MEL}_{\mathrm{CC\text{-}L}}(B) = MFE_{\mathrm{LF}} \cdot \left(\frac{1+\left(\frac{B}{B_{\mathrm{LF}}}\right)^{\alpha}\cos\left(\frac{\pi\alpha}{2}\right)}{1+2\left(\frac{B}{B_{\mathrm{LF}}}\right)^{\alpha}\cos\left(\frac{\pi\alpha}{2}\right)+\left(\frac{B}{B_{\mathrm{LF}}}\right)^{2\alpha}} - 1 \right) + MFE_{\mathrm{HF}} \cdot \left(\frac{B^2}{(B+B_{\mathrm{HF}})^2} \right)$
